# Moderating role of supervisor support in the association between job demands and distress: a mixed-effects analysis in a population-based cohort study

**DOI:** 10.1136/bmjopen-2025-111512

**Published:** 2026-05-12

**Authors:** H A M Lettinga, K I Proper, M F van Wier, S E Kramer, S H van Oostrom, J R Anema

**Affiliations:** 1 Department of Behaviour and Health, Centre for Prevention, Lifestyle and Health, National Institute for Public Health and the Environment (RIVM), Bilthoven, The Netherlands; 2 Department of Public and Occupational Health, Amsterdam UMC, Vrije Universiteit Amsterdam, Amsterdam Public Health Research Institute, Amsterdam, The Netherlands; 3 Department of Otolaryngology-Head and Neck Surgery, Ear & Hearing, Amsterdam UMC, Vrije Universiteit Amsterdam, Amsterdam Public Health Research Institute, Amsterdam, The Netherlands

**Keywords:** Occupational Stress, Job Satisfaction, Social Support, Health Workforce, Workplace, MENTAL HEALTH

## Abstract

**Abstract:**

**Objectives:**

To study the association between job demands and distress among working adults and to test whether perceived supervisor support moderates this relationship.

**Design:**

Mixed-effects analysis of repeated measures from a population-based cohort study, estimating overall (combined within-person and between-person) associations.

**Setting:**

The Netherlands Longitudinal Study on Hearing (NL-SH), an ongoing Dutch cohort with nationwide recruitment and follow-up including four measurement waves.

**Participants:**

A total of 989 employed individuals (≥12 hours/week) with 1858 observations had complete data on distress, job demands, supervisor support and covariates.

**Primary and secondary outcome measures:**

The dependent variable was distress, measured using the 16-item distress subscale (range 0–32) of the Four-Dimensional Symptom Questionnaire. Job demands and supervisor support were assessed with subscales from the Job Content Questionnaire. Multilevel linear models were used to estimate main and interaction effects, adjusted for age, sex, educational level, hearing impairment, contract type and chronic diseases.

**Results:**

Higher job demands were associated with greater distress (B=0.22, 95% CI (0.17 to 0.27)). Higher supervisor support was associated with lower distress (B=–0.26, 95% CI (–0.38 to –0.15)). The interaction between job demands and supervisor support was statistically significant (B=-0.02, 95% CI (-0.04 to 0.001), p=0.042). Stratified analyses showed that the association between job demands and distress was stronger among employees with low supervisor support (B=0.27, p<0.001) than among those with high support (B=0.13, p=0.008).

**Conclusions:**

Job demands and supervisor support were independently associated with distress. Supervisor support appeared to buffer the impact of job demands, as the association between job demands and distress was stronger among employees reporting low levels of supervisor support. These findings underscore the importance of strengthening supportive supervisor practices, alongside addressing excessive job demands, as integral components of workplace mental health strategies.

**STRENGTHS AND LIMITATIONS OF THIS STUDY**

- Repeated measurements from a large, population-based cohort allowed analysis of job demands, perceived supervisor support and distress in a diverse working population.
- Use of mixed-effects models accounted for the clustering of repeated observations within individuals.
- A comprehensive set of sociodemographic and work-related variables was included to adjust for potential confounding.
- Over-representation of participants with hearing impairment in the Netherlands Longitudinal Study on Hearing may limit generalisability to the broader working population, although analyses were adjusted for hearing status.
- The analytical approach combined data across timepoints, limiting the ability to establish temporal ordering or causal direction.

## Introduction

 Distress is a common experience among employees and is characterised by symptoms such as worry, irritability, tension, fatigue, poor concentration and sleep problems.^1 2^ Although conceptualised as a ‘normal’ psychological response to stress, persistent or severe distress can substantially impair functioning and is associated with adverse occupational outcomes, including reduced productivity (long term), sickness absence and increased risk of developing common mental disorders (such as generalised anxiety disorder or major depression).^1^,^5^ The impact of stress-related illnesses on work participation and the economy is substantial. For example, a nationwide study in the Netherlands including 16 676 employees reported that, between 2015 and 2017, an episode of sick leave due to stress-related illness lasted on average 101 working days, resulting in mean employer costs of €19 151/employee. Burnout was associated with the longest absence (on average 163 working days) and the highest costs (mean €30 770).^6^

The relationship between work and distress is complex. While work can contribute to the well-being of the employee (eg, by offering structure, social connections, purpose and financial stability), it can also be a significant source of strain, particularly when job demands exceed the individual’s coping capacity.^2 7 8^ A review of work-related risk factors for common mental health problems showed that high job demands, such as time pressure, workload or role conflict, are associated with elevated levels of distress.^8^ Next to individual coping capacity, effects of high job demands are thought to be shaped by resources in the work environment, such as the degree of job control and social support by coworkers and the supervisor.^9^,^11^ Among these, supervisors occupy a unique position as they can offer structural emotional support (eg, empathy, understanding)^12^ and instrumental support (eg, practical help, redistribution of tasks or workplace adjustments that may directly reduce job demands),^13^ and thus influence how work is organised.

Previous research has highlighted the importance of supervisor support in promoting occupational well-being, including among employees experiencing distress. Evidence shows that workplace interventions targeting organisational stressors (such as high job demands) are more effective in preventing distress when they move beyond individual coping and address systemic issues.^14^ Supervisor involvement is essential for cultivating a healthier work culture where early signs of distress are recognised and addressed.^14^,^16^ In the context of working with mental health complaints, a realist review showed that mechanisms such as perceived supervisor support, job accommodations and an open, trust-based relationship with the employer are crucial.^7^ The authors emphasised that supportive work environments enhance employees’ capabilities to function, meaning the real opportunities to make use of their skills and coping strategies, which in turn promotes work participation even in the presence of mental health challenges.^7^ Similarly, during sickness, absence related to distress, poor relationships with supervisors are frequently identified as key barriers for return to work, while constructive supervisor support facilitates reintegration.^17 18^ Employees often cite high work pressure and a lack of supportive dialogue around job demands as reasons for long-term absence.^17^ Together, these findings suggest that supervisor support can play a role in the work engagement of employees with distress.

Given the negative consequences of distress for employees, employers and society at large, it is important to understand the conditions under which job demands are most harmful. Identifying whether supervisor support can help employees cope with high job demands is crucial, as this knowledge can guide organisations in creating healthier work environments. Supervisor support fulfils an important role in shaping work conditions and could mitigate the impact of high job demands on distress. The present study, therefore, examines whether perceived supervisor support (hereafter: supervisor support) moderates the association between job demands and levels of distress.

## Methods

### Dataset

Data from the Netherlands Longitudinal Study on Hearing (NL-SH) were used for this study. The NL-SH, initiated in 2006, is an ongoing online prospective cohort study in which participants complete a hearing test and questionnaires at 5-year intervals. The questionnaires concern self-reported hearing status, work status and work-related outcomes, physical and psychosocial health, healthcare usage and lifestyle behaviours.^15^ Eligible participants are adults aged 18–70 years at baseline, with or without hearing impairment, who voluntarily choose to participate (see the protocol study for details^19^). Participants have been recruited continuously through multiple channels, including advertisements in newspapers and magazines, flyers at audiology centres and—most prominently—the nationwide web-based Dutch national hearing test (www.hoortest.nl). Adults who completed this online screening and indicated interest in further participation were referred to the NL-SH study website, where they could register and provide informed consent. As recruitment is based on voluntary participation rather than random sampling, the NL-SH constitutes a convenience sample. For the present study, an anonymised data export was obtained, containing data from the baseline (T0), 5-year (T1), 10-year (T2) and 15-year (T3) measurement waves, collected between October of 2006 and January of 2025. As of 2016, this committee-decided amendments (including follow-up waves) are exempt from ethics approval. Participants included in the current study provided digital informed consent for participation in the NL-SH as well as for sharing their data.

### Participants

For the current study, we included all measurement waves from participants who reported being in paid employment and working more than 12 hours per week at the time of assessment. Participants who owned a business (n=31) and those with incomplete data (n=87, 8.2%) were excluded. Missing data predominantly concerned the Job Content Questionnaire (JCQ) and Four-Dimensional Symptom Questionnaire (4DSQ) scales across waves; missingness in covariates was rare (<3%). Compared with the analytic sample, these 87 excluded participants were older (mean age 54.1 vs 47.5 years), more often highly educated (73.6% vs 64.5%), less often women (60.9% vs 62.6%) and less often reported hearing impairment (46.0% vs 51.4%) at their first eligible measurement (see online supplemental table S1). As missingness mainly concerned the psychosocial work variables and distress scores, and the proportion of excluded participants was below 10%, a complete-case approach was considered acceptable.^20^

**Figure 1:**
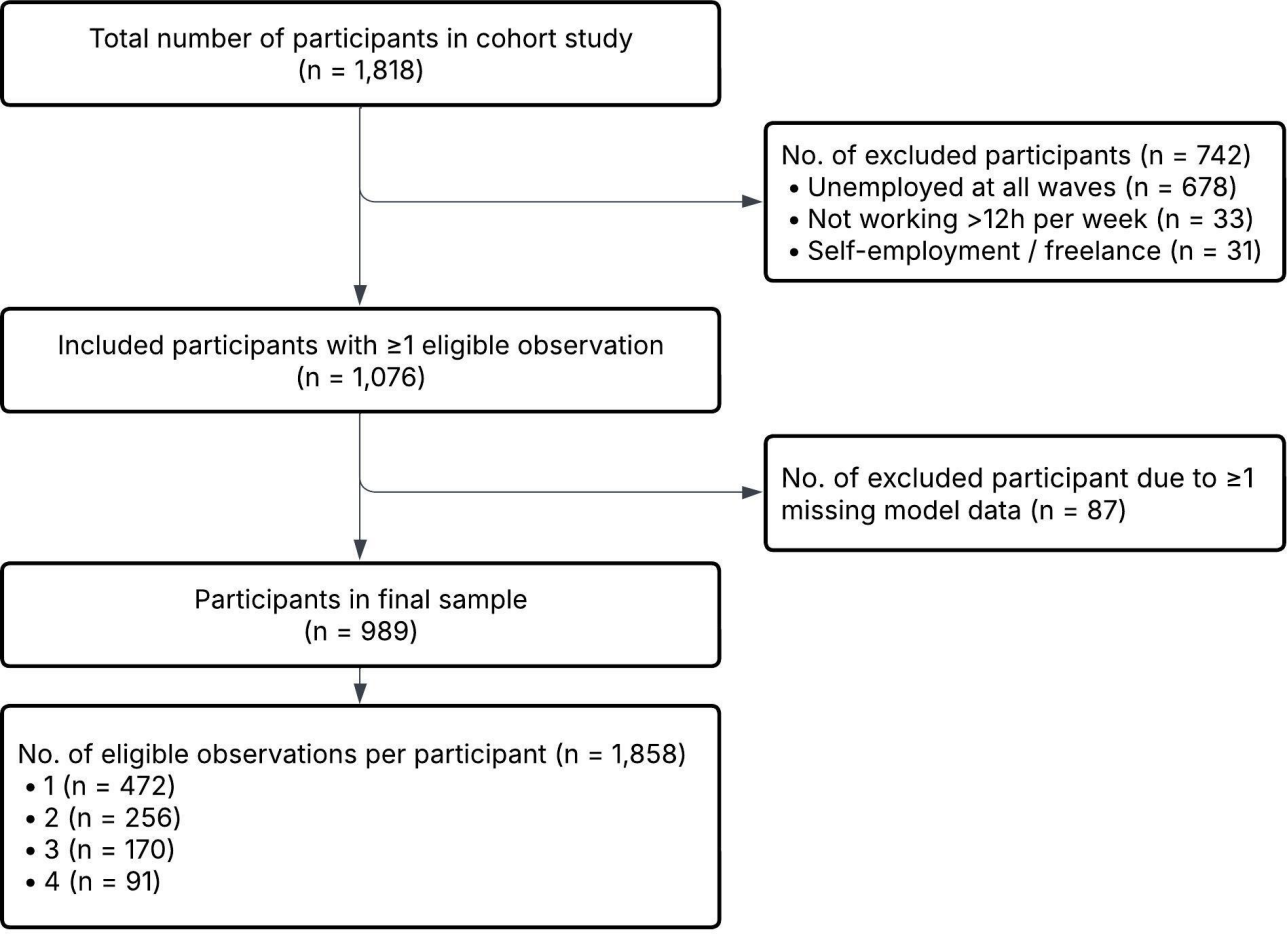
Flow diagram of the selection of the analytic sample. Note: The flow diagram was prepared in accordance with the Strengthening the Reporting of Observational Studies in Epidemiology (STROBE) guidelines.

The final analytic sample consisted of 989 unique employed individuals, contributing a total of 1858 observations. Participants contributed data on one (47.7%), two (25.6%), three (17.2%) or four (9.4%) measurement waves. The flow of participants is illustrated in figure 1.

#### Patient and public involvement

Patient and public involvement in the NL-SH cohort has been described in detail in the cohort profile.^19^ A patient advisory board consisting of individuals with hearing loss has been involved since 2012 in identifying research gaps and refining follow-up questionnaires.

### Variables

#### Distress

The dependent variable distress was measured using the validated Distress subscale of the Dutch 4DSQ,^1 21^ which asks respondents to rate their experiences over the past week. The subscale consists of 16 items (eg, ‘Over the past week, did you feel tense?’), each answered on a 5-point Likert scale (no, sometimes, regularly, often, very often or constantly). Following the scoring guidelines, the Likert ratings are categorised into three levels (0=‘no’, 1=‘sometimes’, 2=‘regularly or more often’). Item scores are summed, resulting in total scores ranging from 0 to 32. Higher scores indicate more severe distress. Scores above 10 are considered moderately elevated, while scores above 20 reflect high levels of distress.^21^

#### Job demands

Job demands were assessed with the 5-item psychological job demands subscale of the validated Dutch version of the JCQ. Respondents were asked to indicate the extent to which they agreed with statements about their work (eg, ‘I have enough time to get the job done’). Items were rated on a 4-point Likert scale (strongly disagree, disagree, agree and strongly agree), representing item scores from 1 to 4. Scores for the subscales were calculated according to the formulae described in the JCQ user guide.^22^ The subscale score ranges from 12 to 48, with higher scores reflecting greater perceived mental workload and time pressure.

#### Supervisor support

Supervisor support was assessed with the 4-item supervisor support subscale of the Dutch JCQ.^22^ Respondents rated their agreement with statements about their supervisor (eg, ‘My supervisor pays attention to what I am saying’) on the same 4-point Likert scale. The subscale score ranges from 4 to 16, with higher scores indicating stronger perceived support from one’s direct supervisor.^22^

#### Covariates/confounders

Potential confounders were selected based on theoretical considerations and prior empirical findings. Demographic characteristics (age, sex and educational level) were included, as these are commonly associated with distress.^23 24^ Educational level was assessed by asking participants to indicate their highest completed level of education, and categorised as low (no formal education to lower vocational training), medium (intermediate general to upper secondary education) or high (higher vocational to postacademic education).^25^

In addition, work-related variables were included. Contract type (permanent vs temporary) and the presence of chronic diseases (yes/no) were also included as both have been shown to influence work-related stress outcomes.^25 26^ Chronic diseases were defined as reporting at least one disease from an extended checklist of chronic diseases, derived from a national health questionnaire of Statistics Netherlands.^27^ Job control, another subscale of the JCQ, was not included as a covariate, as it has been hypothesised to act as a mediator in the association between job demands, supervisor support and distress,^9 11 28^ and adjusting for a mediator may lead to overadjustment when estimating the overall association.

Given the origin of the NL-SH as a cohort focused on hearing, individuals with hearing impairment were over-represented compared with the general working population. Because hearing impairment has been linked to adverse psychosocial work outcomes, a binary indicator of any hearing impairment (yes/no) was included to account for potential confounding. For the current study, hearing impairment was based on self-reported hearing status. Participants were classified as having a hearing impairment if they reported any of the following: conductive hearing loss (eg, eardrum or ossicular chain problems), sensorineural hearing loss, mixed hearing loss, hearing loss due to Ménière’s disease, hearing loss of unknown type or unilateral deafness (with or without additional hearing loss in the other ear). Participants who reported no hearing problems were classified as having no impairment.

#### Statistical analyses

Intercept-only linear mixed-effects models were first estimated to calculate intraclass correlation coefficients (ICCs), quantifying the proportion of variance in distress, job demands and supervisor support attributable to between-person versus within-person differences. Subsequently, a series of two-level linear mixed-effects models were estimated to examine the overall association between job demands, supervisor support and distress, while accounting for repeated measurements (level 1) nested within individuals (level 2). Continuous independent variables (job demands, supervisor support and age) were centred at the grand mean prior to analysis to facilitate interpretation of associations and interaction terms. Because independent variables were centred at the grand mean, the coefficients reflect a combination of between-person differences and within-person variation over time, rather than pure within-person change effects. As an additional analysis, job demands and supervisor support were decomposed into within-person deviations from each individual’s mean and between-person means (grand-mean centred), after which main and interaction models were re-estimated including both components to report within-person and between-person coefficients separately. Analyses were conducted in R V.4.4.0^29^ using the lme4 package for mixed-effects modelling^30^ and ggeffects for visualisation.^31^

To evaluate the potential moderating role of supervisor support, models were specified with both main effects and interaction terms. Model 1 included job demands and supervisor support as fixed effects. In model 2, the interaction term between job demands and supervisor support was added. Wald confidence intervals (95% CI) were used to quantify the precision of all fixed effect estimates. All models were adjusted for age, sex, education level, hearing impairment, chronic diseases and contract type.

Multicollinearity was assessed using variance inflation factors (VIFs) based on an equivalent linear model and the full model including interaction terms. All VIFs were below 1.3, indicating no concerns regarding multicollinearity between the psychosocial work variables.

The distress score showed a moderately right-skewed distribution, with most scores clustering at the lower end of the scale. Residuals were approximately normally distributed, although residual plots indicated moderate heteroscedasticity, which after consultation with a statistician was not considered problematic for the chosen model.

## Results

### Study population

Sample characteristics were calculated at the first eligible measurement occasion for each participant, defined as the first timepoint at which they met the inclusion criteria (ie, paid employment and working ≥12 hours/week). The average participant was 47.5 years old (SD=11.4), and 619/989 were female (62.6%). Most participants were highly educated (64.5%) and held a permanent contract (82.3%). Approximately 56% reported having at least one chronic disease, and 51% reported a hearing impairment. See table 1 for all characteristics.

**Table 1:** Characteristics of the study population at first measurement

Variable	N	Mean (SD)	%
Age (years)	989	47.5 (11.4)	–
Sex			
Male	370		37.4
Female	619		62.6
Educational level			
Low	84		8.5
Intermediate	267		27.0
High	638		64.5
Contract type			
Permanent	814		82.3
Temporary	175		17.7
Hearing impairment			
Yes	508		51.4
No	481		48.6
Chronic diseases			
Yes	550		55.6
No	439		44.4
Distress	989	8.0 (6.8)	–
Job demands	989	30.7 (5.6)	–
Supervisor support	989	11.4 (2.5)	–

Distress scale range: 0–32. Job demands: 12–48. Supervisor support: 4–16.

#### Intraclass correlations

According to the intercept-only models, the ICC for distress was 0.55 (95% CI 0.49 to 0.60), meaning that 55% of the variance in distress was explained by differences between persons. The ICC for job demands was 0.52 (95% CI 0.45 to 0.60), and the ICC for supervisor support was 0.30 (95% CI 0.21 to 0.36), indicating that a substantial proportion of variance in these measures was attributable to between-person rather than within-person differences.

#### Associations between job demands and supervisor support with distress

Higher job demands were associated with greater distress (B=0.22, 95% CI (0.17 to 0.27)). In contrast, higher supervisor support was associated with lower distress (B=–0.26, 95% CI (–0.38 to –0.15)) (model 1, table 2).

**Table 2:** Associations between job demands, supervisor support and distress, adjusted for age, sex, education, hearing impairment, chronic diseases and contract type

Model	Determinant	B	Lower 95% CI	Upper 95% CI	SE	P value
Model 1: main effects	Job demands	0.22	0.17	0.27	0.03	
	Supervisor support	–0.26	–0.38	–0.15	0.06	
Model 2: interaction	Job demands	0.22	0.16	0.27	0.03	
	Supervisor support	–0.26	–0.38	–0.15	0.06	
	Job Demands×Supervisor Support	–0.02	–0.04	–0.001	0.01	0.042
Stratified analysis: low supervisor support (≤ –1 SD)	Job demands	0.27	0.17	0.38	0.05	
High supervisor support (≥ +1 SD)	Job demands	0.13	0.03	0.23	0.05	0.008

Beta’s and 95% CIs are presented. Model 2 additionally includes the interaction between job demands and supervisor support. Stratified analyses show estimates at low (–1 SD) and high (+1 SD) levels of supervisor support.

#### Moderating effect of supervisor support

**Figure 2:**
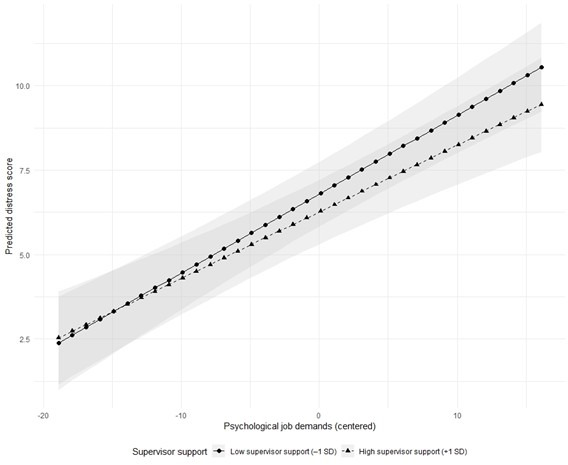
Interaction between job demands and supervisor support in relation to distress. Predicted 4DSQ distress scores (range 0–32) from model 2 are shown across the observed range of centred JCQ job demands (raw scale range 12–48) and were centred at the grand mean before analysis. Lines represent estimated distress at low (–1 SD) and high (+1 SD) levels of JCQ supervisor support (range 4–16). Predicted values are adjusted for: age (set at the sample mean), sex (male), educational level (intermediate), hearing status (no impairment), chronic disease (no) and contract type (permanent). Shaded areas indicate 95% CIs. Predicted values are plotted only within the observed range of centred job demands (–18.9 to +17.1). 4DSQ, Four-Dimensional Symptom Questionnaire; JCQ, Job Content Questionnaire.

The interaction between job demands and supervisor support was statistically significant (B=–0.02, 95% CI (–0.04 to –0.001), p=0.042). Figure 2 visualises the estimated distress levels across the observed range of centred job demands for low (–1 SD) and high (+1 SD) supervisor support, based on model 2, adjusted for age, sex, education, hearing impairment, chronic diseases and contract type. This indicates that higher supervisor support mitigated the positive association between job demands and distress.

As the interaction was significant, an exploratory stratified analysis by supervisor support (–1 SD and +1 SD from the mean) was performed. The association between job demands and distress was stronger among employees with low supervisor support (B=0.27, p<0.001) than among those with high supervisor support (B=0.13, p=0.008), consistent with the observed interaction effect (figure 2). Because supervisor support was centred at the grand mean, the main effect of job demands in model 2 represents the simple slope at the mean level of supervisor support. Simple slopes at low (–1 SD) and high (+1 SD) supervisor support are presented with 95% CIs and p-values.

To facilitate interpretation of the magnitude of the interaction effect, predicted distress scores were calculated for combinations of low (–1 SD) and high (+1 SD) job demands and supervisor support. The predicted distress score ranged from 6.4 among employees with low job demands and high supervisor support to 10.1 among those with high job demands and low supervisor support, approaching the threshold for moderately elevated distress (score >10).

#### Decomposition of within-person and between-persons effects

ICCs showed that a substantial proportion of variance in job demands and supervisor support was between persons (52% and 30%, respectively). When job demands and supervisor support were decomposed into within-person and between-person components, both higher demands and lower support were associated with higher distress at both levels. However, the moderating effect of supervisor support was primarily driven by between-person differences. The interaction between person-level job demands and person-level supervisor support was statistically significant (B = −0.03, p=0.029), whereas the within-person interaction was not (p=0.11). Full model coefficients are shown in online supplemental table S2).

## Discussion

### Main findings

In this study, higher job demands were associated with greater distress, whereas employees who perceived more supervisor support tended to report lower distress. In addition, supervisor support appeared to mitigate the positive association between job demands and distress. Although the magnitude of this moderating effect was modest, the findings suggest that supervisor support may function as a protective resource in demanding work contexts.

### Comparison with the literature

Our findings align with a substantial body of evidence showing that both high job demands and low supervisor support are associated with poorer mental health outcomes, including distress, burnout and reduced work ability.^2 4 16^ Prior studies have shown that supportive supervisors can contribute to improved employee well-being by providing emotional support, facilitating workload adjustments and creating a more inclusive work climate.^7 14 16 32^ For example, employees reporting high supervisor support experienced lower exhaustion and cynicism in a high-demand context during organisational change.^33^ However, systematic reviews of models describing the interplay between job demands, job control and social support^9^ suggest that moderation effects of supervisor support are often inconsistent, varying by study design, population and operationalisation of the determinants.

A further challenge in comparing findings across studies is the heterogeneity in terminology and outcome definitions. Terms such as distress, work-related distress, burnout, stress-related complaints and common mental disorders are frequently used interchangeably, although they do not necessarily refer to the same constructs and are not always grounded in formal diagnostic criteria. In the present study, a validated instrument was used to capture non-specific distress, rather than a clinical diagnosis. However, there is currently no coordinated or standardised diagnostic or management framework to distinguish between distress and other related concepts. This conceptual variability complicates direct comparisons and may partly explain inconsistent evidence for the moderating role of supervisor support.

Our study adds to this literature by demonstrating a moderating effect of supervisor support on the association between job demands and distress in a general working population sample. Although the interaction effect was statistically significant, the observed differences in distress levels between employees with low and high supervisor support remained modest. Still, these differences became more pronounced at higher levels of job demands. For instance, when job demands were high, predicted distress exceeded the threshold for moderate symptoms among those with low support, whereas it remained below that threshold among those with high support.^21^ While such differences may appear small in absolute terms, they can still be meaningful at the population level—particularly if supportive supervision helps prevent distress among employees exposed to high job strain. This is consistent with previous research on psychosocial work exposures, where effect sizes tend to be modest but may nonetheless have implications for workforce health and targeted prevention.^8^ Additional analyses indicated that the moderating effect of supervisor support was primarily driven by between-person differences, suggesting that stable relational or organisational factors may underlie the observed moderation.

### Strengths and limitations

A key strength of this study is the use of data from the NL-SH cohort, which includes a large, diverse sample of working adults and rich data on psychosocial work factors and health outcomes. A comprehensive set of covariates was adjusted for, including sociodemographic and health-related variables, reducing the risk of confounding. In addition, exploratory effect modification analyses indicated that the association between job demands and distress was stronger among employees with low supervisor support than among those with high supervisor support.

Several limitations should be noted. First, although the NL-SH is a longitudinal study with repeated measures, our analyses combined within-person and between-person variation into overall estimates and did not explicitly model temporal ordering. As such, the analyses do not allow conclusions about the direction of the observed associations. Moreover, because all variables were assessed by self-report at the same time point, higher distress may also influence how employees perceive their work environment, for example, by increasing perceived job demands or lowering perceived supervisor support. This potential reverse causation cannot be ruled out given the design. This choice was made to retain as much data as possible, as the 5-year intervals between measurement waves are relatively long for identifying short-term fluctuations in supervisor support or work conditions. At the same time, longer intervals may better capture structural changes such as job transitions or sustained alterations in working conditions. More frequent assessments, ideally complemented with information on job and supervisor changes, would allow for a more detailed understanding of how job demands, supervisor support and distress evolve over time. Second, the NL-SH cohort includes both normally hearing and hearing-impaired participants, but the latter are over-represented compared with the general Dutch population. In mid-life, the prevalence of hearing problems in the general population is estimated at ~13%,^34^ whereas in the current sample about half of the participants reported hearing problems. Although analyses were adjusted for hearing status, this imbalance may still limit generalisability to the broader working population, as the sample does not reflect the distribution of hearing ability in the general workforce. Third, information on job accommodations related to distress (eg, workload adjustments, task redistribution or flexibility in work scheduling) was not available in this study. Such accommodations may influence how employees experience job demands and distress. Evidence from employees with a diagnosed mood disorder suggests that unmet needs for work accommodations related to mental health are associated with poorer workplace outcomes, such as lower job satisfaction and higher turnover intentions.^35^ Because job accommodations were not assessed, it is unclear whether part of the effect of supervisor support reflects its role in enabling workplace adjustments. This may have influenced the strength of the observed associations, particularly if accommodations helped reduce distress in high-demand contexts. Future studies could focus on job accommodations related to distress to better understand their potential role alongside supervisor support. Fourth, the measurement of psychosocial work factors may have underestimated moderation effects. The JCQ supervisor support subscale mainly reflects emotional support and does not capture instrumental support, while our job demands measure did not differentiate between types of demands that may elicit different kinds of support needs. According to the ‘matching principle’, moderation is most likely when the type of support corresponds to the type of need.^9^ For participants with moderate to severe hearing loss in this cohort, such stressors are often instrumental in nature (eg, need for acoustic adjustments, assistive listening devices or adapted meeting practices). Because these aspects were not assessed, moderation effects may have been underestimated. Instruments such as the Support Appraisal for Work Stressors Inventory,^36^ which distinguishes between emotional and instrumental supervisor support, could provide a more nuanced assessment in future studies. In the present dataset, no separate measure of instrumental supervisor support was available, which precluded additional secondary analyses using alternative operationalisations. Similarly, psychological job demands were assessed as an aggregate construct and did not allow separate examination of distinct demand types (eg, quantitative vs cognitive workload). This means that potential heterogeneity in the buffering effect across different kinds of job demands could not be examined. Clarifying both types of support and differentiating between specific job demands would help to identify the conditions under which supervisor support is most effective.

### Implications

These findings highlight that the way supervisors support their employees can make a difference for how job demands are experienced, underlining the importance of the social work environment for employee well-being. Investing in supervisors’ ability to provide both emotional and instrumental support therefore represents a concrete and promising target for workplace interventions. Such programmes have been shown to reduce stress, improve work engagement and prevent sickness absence.^37 38^ According to the matching principle, stronger buffering effects may be expected when the type of support more closely corresponds to the type of need, suggesting that interventions should be tailored to the specific occupational context rather than applying a one-size-fits-all approach. Participatory approaches are one example of such interventions, in which supervisors and employees jointly identify and address work barriers tailored to the situation at hand. Participatory approaches, if implemented correctly, have been shown to lead to reductions in job demands and improvements in job satisfaction in employees with distress,^39^ and a faster return-to-work in a subset of employees on sickness absence with distress.^40^ If such approaches do not lead to the desired result, and the supervisor or employee identify signs of increasing distress or a risk of sickness absence, involving an occupational physician may help to facilitate timely support and, where appropriate, workplace adjustments.

## Conclusions

This study indicates that higher job demands are associated with greater distress, whereas employees who perceive more supervisor support report lower levels of distress. Supervisor support appears to buffer the negative impact of job demands, as the association between job demands and distress was stronger among employees reporting low levels of supervisor support. The findings underscore that besides addressing high workplace demands, investing in supervisors’ skills and qualities to support employees in an appropriate manner should be an integral part of workplace health strategies. Facilitating joint problem-solving between supervisors and employees may further strengthen the protective role of supervisor support.

## Supplementary material

10.1136/bmjopen-2025-111512 online supplemental table 1

10.1136/bmjopen-2025-111512 online supplemental table 2

## Data Availability

Data are available upon reasonable request.
