# Supplementary material for: Moderating role of supervisor support in the association between job demands and distress: a mixed-effects analysis in a population-based cohort study
Source: BMJ Open. 2026 May 12;16(5):e111512. doi: 10.1136/bmjopen-2025-111512 (PMC13182364; doi:10.1136/bmjopen-2025-111512)
Supplement: online supplemental table 1 [file bmjopen-16-5-s001.docx]

Supplementary Table S1. *Comparison of included and excluded participants (first eligible measurement)*

| Characteristic | Analytic sample (n = 989) | Excluded participants (n = 87) |
| --- | --- | --- |
| Mean age, (years [SD]) | 47.5 [11.4] | 54.1 [10.8] |
| Female (%) | 62.6 | 60.9 |
| High education (%) | 64.5 | 73.6 |
| Hearing impairment (%) | 51.4 | 46.0 |
