# Supplementary material for: Moderating role of supervisor support in the association between job demands and distress: a mixed-effects analysis in a population-based cohort study
Source: BMJ Open. 2026 May 12;16(5):e111512. doi: 10.1136/bmjopen-2025-111512 (PMC13182364; doi:10.1136/bmjopen-2025-111512)
Supplement: online supplemental table 2 [file bmjopen-16-5-s002.docx]

Supplementary Table S2. *Within- and between-person associations between job demands, supervisor support, and distress, adjusted for age, sex, education, hearing impairment, chronic diseases, and contract type.*

| **Model** | **Determinant** | **B** | **Lower 95% CI** | **Upper 95% CI** | **SE** | **p-value** |
| --- | --- | --- | --- | --- | --- | --- |
| **Model 1: Main effects** | **Within-person effects** |  |  |  |  |  |
|  | Job demands | 0.19 | 0.11 | 0.26 | 0.040 | <0.001 |
|  | Supervisor support | −0.15 | −0.31 | 0.00 | 0.078 | 0.051 |
|  | **Between-person effects** |  |  |  |  |  |
|  | Job demands | 0.25 | 0.18 | 0.32 | 0.037 | <0.001 |
|  | Supervisor support | −0.41 | −0.58 | −0.24 | 0.088 | <0.001 |
|  |  |  |  |  |  |  |
| **Model 2: Interaction** | **Within-person effects** |  |  |  |  |  |
|  | Job demands | 0.19 | 0.11 | 0.26 | 0.040 | <0.001 |
|  | Supervisor support | −0.15 | −0.30 | 0.01 | 0.079 | 0.061 |
|  | Job demands × Supervisor support | −0.05 | −0.11 | 0.01 | 0.031 | 0.108 |
|  | **Between-person effects** |  |  |  |  |  |
|  | Job demands | 0.24 | 0.17 | 0.31 | 0.037 | <0.001 |
|  | Supervisor support | −0.41 | −0.58 | −0.24 | 0.088 | <0.001 |
|  | Job demands × Supervisor support | −0.03 | −0.06 | −0.003 | 0.014 | 0.029 |
