# Supplementary material for: Moderating role of supervisor support in the association between job demands and distress: a mixed-effects analysis in a population-based cohort study
Source: BMJ Open. 2026 May 12;16(5):e111512. doi: 10.1136/bmjopen-2025-111512 (PMC13182364; doi:10.1136/bmjopen-2025-111512)
Supplement: Supplementary file 3 [file bmjopen-2025-111512.reviewer_comments.pdf]

PEER REVIEW HISTORY

BMJ Open publishes all reviews undertaken for accepted manuscripts. Reviewers are asked to complete a checklist review form and are provided with free text boxes to elaborate on their assessment. These free text comments are reproduced below.

ARTICLE DETAILS

Title (Provisional)

The Moderating Role of Supervisor Support in the Association Between Job Demands and Distress: A Mixed-Effects Analysis in a Population-Based Cohort Study

Authors

Lettinga, Hanneke A. M.; Proper, Karin; van Wier, Marieke F.; Kramer, Sophia E.; van Oostrom, Sandra H; Anema, Johannes

---

VERSION 1 - REVIEW

**Reviewer**	**1**
**Name**	**Vitturi, Bruno**
**Affiliation**	**University of Genoa, DISSAL**
**Date**	**17-Oct-2025**
**COI**	**None**

I had the pleasure of reading your manuscript, "The Moderating Role of Supervisor Support in the Association Between Job Demands and Distress: A Mixed-Model Analysis." The paper is interesting and well-written, and the topic certainly deserves greater attention from the medical community. However, I would like to highlight a few important points that should be addressed.

Job accommodations:

Do the authors have any information regarding whether participants received any type of job accommodation? This aspect is highly relevant. Even if such data are not available, it would be valuable to include a short paragraph in the Discussion section addressing this issue. For instance, do the authors believe that job accommodations might influence the outcomes observed in this study?

Role of the medical doctor:

I suggest that the authors elaborate on the role of medical doctors in preventing and managing work-related distress. Is there indeed a defined role? If so, what type of doctor should take the lead in such cases?

Distress versus work-related stress:

Workplace distress may, in fact, be considered synonymous with the well-known occupational condition “work-related stress.” Unfortunately, there is still a lack of coordinated efforts to standardize the diagnostic approach and management of this condition. This important gap should be highlighted in the discussion.

Relevance of recent literature:

Lastly, the authors should not omit the article doi: 10.1007/s10926-023-10149-9 from the discussion. Multiple sclerosis provides an excellent example of a condition that causes numerous invisible symptoms, including distress. That study also demonstrates how distress is associated with other relevant outcomes, such as mood disorders, motivation to remain at work, quality of life, and cognitive functioning. This evidence should be integrated into the discussion.

**Reviewer**	**2**
**Name**	**Poursadeqiyan, Mohsen**
**Affiliation**	**Ardabil University of Medical Sciences**
**Date**	**22-Nov-2025**
**COI**	**None**

This critical review provides an overview and could serve as a guide for authors or reviewers to improve the manuscript before publication.

Introduction

Weaknesses: It may contain excessive detail on general concept before specifically addressing the target population. A more focused narrative might improve clarity. You must most search for new related references in this field such as fundamental study:

doi: 10.3233/WOR-210283.

DOI: 10.3233/WOR-213648

DOI: 10.3233/WOR-203368

doi: 10.1080/10803548.2016.121699

PMID: 29308385

DOI: 10.7860/JCDR/2019/37929.12482

at the continue 10 major and minor comment. : Major Comments

10 Major Comments (critical issues that could affect validity, interpretation, or publishability)

1. Causality and temporal ordering

• Issue: The authors acknowledge that the design is longitudinal with repeated measures but state that analyses combine within- and between-person variation and do not establish temporal ordering.

• Impact: This limits causal inference about whether job demands lead to distress and whether supervisor support changes this trajectory.

• Recommendation: Re-analyze with a proper longitudinal approach that separates within-person changes over time from between-person differences (e.g., random-intercept cross-lagged panel models, or a two-level lagged model where distress at time t is regressed on job demands and supervisor support at time t-1, with appropriate controls). Consider presenting supplementary analyses that explore directionality.

2. Handling of within- vs between-person effects

• Issue: The manuscript uses two-level linear mixed-effects models but does not clearly distinguish or quantify within-person (time-varying) effects vs between-person (time-invariant) effects for demands and support.

• Impact: Mixing these sources of variance can obscure interpretation of moderation and potentially inflate or obscure interaction effects.

• Recommendation: Explicitly decompose within- and between-person effects (e.g., use person-mean centering for time-varying predictors and include a between-person component). Report both within- and between-person coefficients for the main effects and interaction.

3. Moderation interpretation and clinical/practical significance

• Issue: The interaction term is statistically significant ($p = 0.042$) but the reported effect size is small ($B = -0.02$ on distress).

• Impact: Practically, the buffering effect may be limited; readers may question whether this is meaningful in real-world settings.

• Recommendation: Provide a more interpretable magnitude of buffering (e.g., translate into predicted distress score differences across realistic ranges of job demands and supervisor support). Include effect sizes such as standardized beta or a minimal clinically important difference (if available) for the 4DSQ distress scale.

4. Model specification and potential multicollinearity

• Issue: Although VIFs are reported as <1.3, the manuscript should more fully justify the choice of covariates and the decision to exclude job control as a covariate (despite acknowledging its potential mediation).

• Impact: Omission of key related predictors could bias estimates.

• Recommendation: Consider testing a sensitivity model that includes job control to assess robustness, and discuss mediation vs moderation frameworks more explicitly. Report variance explained (R^2-like metrics) for fixed effects if feasible.

5. Covariate handling and potential confounding

• Issue: Hearing impairment is included as a binary covariate to adjust for overrepresentation, but there may be residual confounding related to hearing loss severity, communication challenges, or related workplace accommodations.

• Impact: Potential residual confounding could bias estimates of the job demands–distress relationship and the moderation by supervisor support.

• Recommendation: If data permit, test whether a more nuanced hearing impairment variable (e.g., severity, use of hearing aids, accommodation status) alters results. At minimum, discuss residual confounding and limitations more explicitly.

6. Generalizability given the sample and context

• Issue: The NL-SH cohort overrepresents hearing-impaired individuals and has a high proportion of highly educated, permanent workers; 51% reported hearing impairment.

• Impact: External validity to the broader Dutch workforce (and other populations) may be limited.

• Recommendation: Provide a more explicit discussion of generalizability and consider weighting or stratified analyses by hearing status if feasible. If not, clearly state the limits in the discussion.

7. Measurement limitations of key constructs

• Issue: Job demands are measured with a five-item psychological demands subscale; supervisor support uses a four-item subscale of the Dutch JCQ. The authors note that the JCQ focuses on emotional support and may miss instrumental support.

• Impact: Measurement limitations could attenuate or misrepresent moderation effects (matching principle).

• Recommendation: Acknowledge and, if possible, conduct secondary analyses using alternative operationalizations (e.g., separate subscales for emotional vs instrumental support, if data exist). Consider discussing SAWSI or other measures for future work.

8. Matching principle and specificity of demands/support

• Issue: The authors discuss the matching principle but the study did not distinguish types of job demands (e.g., quantitative vs cognitive) or types of supervisor support.

• Impact: The moderation may be stronger for certain demands or support types; aggregated measures could mask heterogeneity.

• Recommendation: If data permit, perform exploratory analyses by subgrouping job demands (e.g., time pressure vs workload) and examine whether the buffering effect varies by demand type. Emphasize this as a direction for future work.

9. Handling of missing data and attrition

• Issue: Excluded participants differed on several characteristics; the analysis uses a complete-case approach (n = 989 with 1,858 observations) from an eligible pool of larger size.

• Impact: Potential selection bias and reduced generalizability.

• Recommendation: Describe missing data patterns and consider multiple imputation or mixed-model advantages that handle missingness under MAR. Include a sensitivity analysis comparing complete-case vs imputed results.

10. STROBE/REDCAP-type reporting completeness

• Issue: The manuscript includes many elements well, but reporting could be strengthened in key areas (flow diagram, missing data details beyond the baseline).

• Impact: Reviewers may note gaps in reporting standards.

• Recommendation: Ensure a clear flow diagram with numbers at each stage (eligibility, inclusion, retention) across waves, and provide a table of missing data by variable and wave. Align with STROBE/MG guidelines explicitly in the methods/results sections.

10 Minor Comments (clarifications, formatting, and minor enhancements)

1. Abstract clarity

• Suggestion: Include the study design term (e.g., "longitudinal cohort study with mixed-effects modeling") in the abstract's design line for quick indexing.

2. Consistency of terminology

• Suggestion: Use consistent terminology for distress (4DSQ Distress) and ensure that "distress" vs "stress-related symptoms" aren't conflated in the narrative.

3. Figures and tables readability

• Issue: Some figures reference Model 2 and observed ranges; ensure captions clearly explain what is plotted (axes, units, SD units).

• Suggestion: Add axis labels with real units and annotate effect sizes in the figure or include a supplementary figure with raw data ranges.

4. Reporting of interaction

• Suggestion: Report the simple slopes at representative values of supervisor support (e.g., low, mean, high) with CIs to aid interpretation, in addition to the stratified analysis.

5. Statistical software details

• Note: They used R, lme4, and ggeffects. Consider providing code snippets or linking to an accessible repository (if allowed by data sharing policies) to enhance reproducibility.

6. Data availability and ethics

• Note: Data availability statement is present; ensure that documentation for data access (application process) is precise. Confirm ethics approvals across waves if applicable.

7. Table 1 (descriptive characteristics)

• Suggestion: Include p-values for group comparisons (if any) and/or standardized differences to assess balance, particularly for key covariates.

8. Multicollinearity discussion

• Suggestion: Provide VIFs for the full model (including interaction term). Explain any tolerance concerns or how collinearity was handled when interpreting the interaction.

9. Limitations expansion

• Suggestion: Add a brief note on potential reverse causation (e.g., distress influencing perceived supervisor support or perceived job demands) and how the study design mitigates or cannot mitigate this.

10. Practical implications

• Suggestion: Provide concrete recommendations for workplaces (e.g., supervisor training components, documentation of supportive practices) and outline how organizations might monitor these variables in practice.

• Discussion and Conclusion: A thorough analysis and interpretation of the study findings in the upcoming sections could significantly strengthen the scientific contribution of the article

• use new updated references such as above and below references: in some mental aspect

• doi: 10.32598/hdq.9.4.527.2

• PMCID: PMC9621363 stress DOI: 10.4103/ATMPH.ATMPH_306_17

• DOI: 10.4103/ATMPH.ATMPH_305_17 doi: 10.18502/ijph.v49i10.4699

• doi: 10.3233/WOR-203165 shift work https://acgih.ir/wp-content/uploads/2018/09/988.pdf

• burn out: PMC6169446

• PMCID: PMC8643524 organization aspect http://dx.doi.org/10.3923/rjmsci.2016.307.312

• DOI: 10.4103/jehp.jehp_309_20

• URL: http://rehabilitationj.uswr.ac.ir/article-1-3626-fa.html

• https://www.makhillpublications.co/public/files/published-files/mak-rjas/2016/7-547-553.pdf

• https://makhillpublications.co/files/published-files/mak-rjms/2016/4-307-312.pdf

•

VERSION 1 - AUTHOR RESPONSE

Reviewer 1 comments to author

1. **Job accommodations**

Do the authors have any information regarding whether participants received any type of job accommodation? This aspect is highly relevant. Even if such data are not available, it would be valuable to include a short paragraph in the Discussion section addressing this issue. For instance, do the authors believe that job accommodations might influence the outcomes observed in this study?

Response: Unfortunately, information on job accommodations for employees with distress (e.g., workload adjustments or other workplace adaptations) was not collected in the NL-SH study. We agree that accommodations may influence how employees experience job demands and distress, and we have now explicitly acknowledged this as a limitation and briefly discussed the potential role of job accommodations in the discussion section.

Adjustment in manuscript, page 13, line number 363:
"Third, information on job accommodations related to distress (e.g. workload adjustments, task redistribution or flexibility in work scheduling) was not available in this study. Such accommodations may influence how employees experience job demands and distress. Evidence from employees with a diagnosed mood disorder suggests that unmet needs for job accommodations related to mental health are associated with poorer workplace outcomes, such as lower job satisfaction and higher turnover intentions (33). Because job accommodations were not assessed, it is unclear whether part of the effect of supervisor support reflects its role in enabling workplace adjustments. This may have influenced the strength of the observed associations, particularly if accommodations helped reduce distress in high-demand contexts. Future studies could focus on job accommodations related to distress to better understand their potential role alongside supervisor support."

2. **Role of the medical doctor**

I suggest that the authors elaborate on the role of medical doctors in preventing and managing work-related distress. Is there indeed a defined role? If so, what type of doctor should take the lead in such cases?

Response: Health professionals, especially occupational physicians in the Dutch context, play an important role in preventing and managing work-related stress. However, the aim of the present study was to examine the association between job demands and distress among working adults, and to test whether perceived supervisor support moderates this relationship. We did not focus on clinical care pathways or on the roles of specific medical disciplines. Therefore, we believe it would be beyond the scope of the manuscript to provide a

detailed discussion of which doctor should take the lead.
To acknowledge this point, we have added a short sentence in the Discussion noting that physicians working at the interface of work and health (such as occupational physicians) may contribute to early recognition of distress and to supporting communication about work demands when relevant.

Adjustment in manuscript, page 14, line number 410:
"If such approaches do not lead to the desired result, and the supervisor or employee identify signs of increasing distress or a risk of sickness absence, involving an occupational physician may help to facilitate timely support and, where appropriate, workplace adjustments."

3. **Distress versus work-related stress**

Workplace distress may, in fact, be considered synonymous with the well-known occupational condition "work-related stress." Unfortunately, there is still a lack of coordinated efforts to standardize the diagnostic approach and management of this condition. This important gap should be highlighted in the discussion.

Response: Terminology in this field is indeed not standardized and concepts such as distress, work-related distress, burnout, stress-related complaints and common mental disorders are often used interchangeably. We therefore did discuss in our manuscript that the conceptual variability may complicate comparisons across studies and have now expanded and clarified this further in the discussion. In particular, we make explicit that in the present study we assessed distress using a validated instrument that captures non-specific distress rather than a clinical diagnosis, and that there is currently no standardized diagnostic or management framework for distinguishing between work-related stress, distress and related concepts. This lack of standardization may further contribute to heterogeneity in reported findings.

Adjustment in manuscript, page 11, line number 309:
"A further challenge in comparing findings across studies is the heterogeneity in terminology and outcome definitions. Terms such as distress, work-related distress, burnout, stress-related complaints, and common mental disorders are frequently used interchangeably, although they do not necessarily refer to the same constructs and are not always grounded in formal diagnostic criteria. In the present study, a validated instrument was used to capture non-specific distress, rather than a clinical diagnosis. However, there is currently no coordinated or standardized diagnostic or management framework to distinguish between distress and other related concepts. This conceptual variability complicates direct comparisons and may partly explain inconsistent evidence for the moderating role of supervisor support."

4. **Relevance of recent literature**

Lastly, the authors should not omit the article doi: 10.1007/s10926-023-10149-9 from the discussion. Multiple sclerosis provides an excellent example of a condition that causes numerous invisible symptoms, including distress. That study also demonstrates how distress is associated with other relevant outcomes, such as mood disorders, motivation

to remain at work, quality of life, and cognitive functioning. This evidence should be integrated into the discussion.

Response: We carefully considered the proposed article. However, the manuscript focuses on a disease-specific clinical population (individuals with multiple sclerosis), whereas the present study aims to examine work-related determinants of distress in a general working-age population sample. Although the NL-SH cohort includes both normally-hearing and hearing-impaired participants, hearing status is not the focus of the present analyses. Our study therefore does not take a disease-specific clinical perspective. For this reason, we believe that including the suggested reference would shift the focus of the discussion away from the primary aim of the manuscript. We therefore decided not to add this reference, while acknowledging that it represents a valuable contribution to the clinical literature on mental health in people with multiple sclerosis.

Reviewer 2

1. **Introduction focus and references**

It may contain excessive detail on general concept before specifically addressing the target population. A more focused narrative might improve clarity. You must most search for new related references in this field such as fundamental study:
doi: 10.3233/WOR-210283
DOI: 10.3233/WOR-213648
DOI: 10.3233/WOR-203368
doi: 10.1080/10803548.2016.121699
PMID: 29308385
DOI: 10.7860/JCDR/2019/37929.12482

Response: Thank you for the additional literature suggestions. We carefully reviewed the proposed references. Several of these articles provide valuable insights within specific occupational, clinical, or regional contexts (for example, nursing, shift work, or burnout prevalence in selected populations). However, their focus and scope differ from that of the present study. Many of the suggested references primarily address prevalence estimation, context-specific stressors, or single-occupation samples, whereas our manuscript examines associations between psychosocial work factors—specifically job demands and supervisor support—and distress in a general working-age population.

We therefore concluded that the suggested literature does not directly inform the research question or analytical framework of the current study. In the revised introduction, we have strengthened the narrative by incorporating recent and relevant literature on psychosocial work factors and mental health that aligns closely with our study aims. In line with the editor's guidance to include only references that are directly relevant to the manuscript, we have chosen not to add the proposed references.

2. **Causality and temporal ordering**

Issue: The authors acknowledge that the design is longitudinal with repeated measures but state that analyses combine within- and between-person variation and do not establish temporal ordering.

Impact: This limits causal inference about whether job demands lead to distress and whether supervisor support changes this trajectory.
Recommendation: Re-analyze with a proper longitudinal approach that separates within-person changes over time from between-person differences (e.g., random-intercept cross-lagged panel models, or a two-level lagged model where distress at time t is regressed on job demands and supervisor support at time t–1, with appropriate controls). Consider presenting supplementary analyses that explore directionality.

Response: We agree that separating within-person change from between-person differences and modelling temporal ordering can be highly informative. However, the NL-SH data are collected at approximately five-year intervals, which makes it difficult to specify meaningful lag structures for short-term psychosocial exposures or distress and limits the interpretability of lagged or cross-lagged models in this context (1). Therefore, our analyses were designed to estimate overall associations between job demands, supervisor support and distress in a working-age population, while accounting for clustering of repeated observations within individuals.

We therefore did not re-analyze the data using lagged or cross-lagged approaches, as these would imply a different conceptual and analytical framework than intended in the present manuscript. Instead, we have clarified this more explicitly in the Methods, emphasizing that the mixed-effects models combine within- and between-person variation and that the results should not be interpreted as causal or as evidence of temporal ordering.

Adjustment in manuscript, page 7, line number 216:
"Because independent variables were centered at the grand mean, the coefficients reflect a combination of between-person differences and within-person variation over time, rather than pure within-person change effects."

3. **Handling of within- vs between-person effects**

Issue: The manuscript uses two-level linear mixed-effects models but does not clearly distinguish or quantify within-person (time-varying) effects vs between-person (time-invariant) effects for demands and support.
Impact: Mixing these sources of variance can obscure interpretation of moderation and potentially inflate or obscure interaction effects.
Recommendation: Explicitly decompose within- and between-person effects (e.g., use person-mean centering for time-varying predictors and include a between-person component). Report both within- and between-person coefficients for the main effects and interaction.

Response: Following the reviewer's recommendation, we conducted an additional decomposition analysis. We estimated both main effects and interactions for the within- and between-person components. The results indicate that the buffering effect that we found in the overall analysis is primarily driven by between-person differences (between-

person interaction B = −0.03, p = 0.03), whereas the within-person interaction was not statistically significant (p = 0.11). These findings are now reported as additional analyses (Supplementary Table S2) and are considered in the interpretation.

Adjustment in manuscript:

- Methods, page 7, line number 218: "As an additional analysis, job demands and supervisor support were decomposed into within-person deviations from each individual's mean and between-person person means (grand-mean centered), after which main and interaction models were re-estimated including both components to report within- and between-person coefficients separately."

- Results, page 11, line number 281: "Decomposition of within- and between-persons effects. ICCs showed that a substantial proportion of variance in job demands and supervisor support was between persons (52% and 30%, respectively). When job demands and supervisor support were decomposed into within- and between-person components, both higher demands and lower support were associated with higher distress at both levels. However, the moderating effect of supervisor support was primarily driven by between-person differences. The interaction between person-level job demands and person-level supervisor support was statistically significant (B = −0.03, p = 0.029), whereas the within-person interaction was not (p = 0.11). Full model coefficients are shown in Supplementary Table S2."

- Discussion, page 12, line number 331: "Additional analyses indicated that the moderating effect of supervisor support was primarily driven by between-person differences, suggesting that stable relational or organizational factors may underlie the observed moderation."

- Supplementary table S2

4. Moderation interpretation and clinical/practical significance

Issue: The interaction term is statistically significant (p = 0.042) but the reported effect size is small (B = −0.02 on distress).
Impact: Practically, the buffering effect may be limited; readers may question whether this is meaningful in real-world settings.
Recommendation: Provide a more interpretable magnitude of buffering (e.g., translate into predicted distress score differences across realistic ranges of job demands and supervisor support). Include effect sizes such as standardized beta or a minimal clinically important difference (if available) for the 4DSQ distress scale.

Response: We agree that the interaction effect is small in absolute terms and acknowledge we should give some interpretation on the relevance of this small effect in the discussion section. In line with current epidemiological reporting standards, we present the unstandardized regression coefficients in the results section. To aid interpretation, predicted distress scores across realistic ranges of job demands and supervisor support are already presented using marginal effects plots generated with ggeffects (figure 2), including simple slopes at low (–1 SD) and high (+1 SD) levels of supervisor support. These

figures illustrate that the buffering effect is modest, but that the difference in predicted distress increases as job demands rise. For instance, at a job demands score of +10 (centered), the predicted distress score is approximately 11.0 when supervisor support is low (–1 SD), compared to 9.0 when supervisor support is high (+1 SD). This 2-point difference crosses the threshold for moderately elevated distress (cut-off score = 10), and, although modest in absolute terms, may still be relevant at the population level in terms of prevention and intervention targets.

To aid interpretation of absolute distress levels, the Methods section clarifies that 4DSQ Distress scores above 10 indicate moderately elevated distress and scores above 20 indicate high distress (2, 3). However, a validated minimal clinically important difference has not been established for the 4DSQ Distress scale in general working populations. We therefore refrain from inferring individual-level clinical significance from the observed effect sizes. At the same time, even small differences in mean distress levels may still be relevant at the population level. We have now made this interpretation more explicit in the Discussion.

Adjustment in manuscript, page 12, line number 321:
"Although the interaction effect was statistically significant, the observed differences in distress levels between employees with low and high supervisor support remained modest. Still, these differences became more pronounced at higher levels of job demands. For instance, when job demands were high, predicted distress exceeded the threshold for moderate symptoms among those with low support, whereas it remained below that threshold among those with high support. While such differences may appear small in absolute terms, they can still be meaningful at the population level—particularly if supportive supervision helps prevent distress among employees exposed to high job strain. This is consistent with previous research on psychosocial work exposures, where effect sizes tend to be modest but may nonetheless have implications for workforce health and targeted prevention(8)."

5. **Model specification and potential multicollinearity**

Issue: Although VIFs are reported as <1.3, the manuscript should more fully justify the choice of covariates and the decision to exclude job control as a covariate (despite acknowledging its potential mediation).
Impact: Omission of key related predictors could bias estimates.
Recommendation: Consider testing a sensitivity model that includes job control to assess robustness, and discuss mediation vs moderation frameworks more explicitly. Report variance explained (R^2-like metrics) for fixed effects if feasible.

Response: We agree that careful justification of covariate selection is important in studies of psychosocial work factors and distress. In the present manuscript, potential confounders were selected a priori based on theoretical considerations and previous research linking sociodemographic characteristics, health status, and employment conditions to distress. This rationale is described in the Methods section.

Job control, although available in the dataset, was not included as a covariate because it has been hypothesized to act as a mediator in the association between job demands, supervisor support and distress. Adjusting for a potential mediator may introduce over-adjustment bias and obscure the overall association of interest. We have clarified this more explicitly in the manuscript.

With respect to multicollinearity, we assessed variance inflation factors in an equivalent linear model. All VIFs were <1.3, indicating that intercorrelations between the psychosocial work variables were not a source of instability in the estimates

Adjustment in manuscript, page 6, line number 191: "Job control, another subscale of the JCQ, was not included as a covariate, as it has been hypothesized to act as a mediator in the association between job demands, supervisor support, and distress (9, 11, 28), and adjusting for a mediator may lead to over-adjustment when estimating the overall association."

6. **Covariate handling and potential confounding**

Issue: Hearing impairment is included as a binary covariate to adjust for overrepresentation, but there may be residual confounding related to hearing loss severity, communication challenges, or related workplace accommodations.
Impact: Potential residual confounding could bias estimates of the job demands-distress relationship and the moderation by supervisor support.
Recommendation: If data permit, test whether a more nuanced hearing impairment variable (e.g., severity, use of hearing aids, accommodation status) alters results. At minimum, discuss residual confounding and limitations more explicitly.

Response: We agree that hearing impairment varies in severity and that a dichotomous covariate may not fully capture heterogeneity in communication difficulties or work-related consequences. To examine whether our results were sensitive to a more detailed adjustment for hearing status, we conducted post-hoc sensitivity analyses using objective Speech Reception Threshold (SRT) measures, which were available for 963 of the 989 included participants.

First, we replaced the dichotomous hearing impairment variable with a three-level SRT-based categorical variable (Good / Insufficient / Poor hearing). The estimated main effects of job demands and supervisor support on distress remained virtually unchanged compared with the original models (job demands ß = 0.222 vs 0.220; supervisor support ß = −0.262 vs −0.265). In this model, only the "Poor hearing" category was significantly associated with higher distress (ß = 0.98, p = 0.04), whereas the "Insufficient" category did not differ from "Good hearing" (ß = 0.58, p = 0.26).

We then re-estimated the interaction model including the SRT categories as confounders. The job demands × supervisor support interaction remained similar in direction and magnitude (ß = −0.0166 vs −0.0180), although it no longer reached conventional statistical significance (p = 0.065 vs p = 0.042), probably partly due to the smaller sample size (n =

963 instead of 989) and the additional degrees of freedom introduced by the three-category variable.

Second, we repeated these analyses using continuous SRT scores, additionally adjusting for transducer type. Again, the main effects were essentially unchanged (job demands ß = 0.222; supervisor support ß = −0.257), and the interaction remained similar in magnitude (ß = −0.0173) but became marginally non-significant ($p = 0.054$).

Taken together, these sensitivity analyses indicate that the original conclusions of the study are robust with respect to how hearing status is modelled: the direction and magnitude of both main effects and the interaction are stable, but models that include more detailed hearing adjustment have less statistical power due to reduced sample size and additional parameters. Because hearing impairment is not the primary focus of this manuscript and because inclusion of SRT-based measures would complicate the main models without changing their substantive interpretation, we chose to retain the dichotomous covariate in the manuscript and to report these sensitivity analyses here. If the editor or reviewers consider it useful, we would be happy to include the full results of these sensitivity analyses in a supplementary table.

7. **Generalizability given the sample and context**

Issue: The NL-SH cohort overrepresents hearing-impaired individuals and has a high proportion of highly educated, permanent workers; 51% reported hearing impairment.
Impact: External validity to the broader Dutch workforce (and other populations) may be limited.
Recommendation: Provide a more explicit discussion of generalizability and consider weighting or stratified analyses by hearing status if feasible. If not, clearly state the limits in the discussion.

Response: We agree that external validity should be considered carefully when interpreting these findings. The NL-SH study deliberately oversamples adults with hearing problems, which means that hearing impairment is substantially more prevalent in our sample than in the general Dutch workforce. To account for this, hearing impairment was included as a covariate in all models, but we acknowledge that residual imbalance may still influence generalizability.
We have now discussed this limitation more explicit in the discussion section. In line with the aims of the present study, no weighting or stratified analyses were conducted, as our focus was on estimating associations between job demands, supervisor support, and distress rather than producing population-representative prevalence estimates.

Adjustment in manuscript, page 13, line number 361: "Although analyses were adjusted for hearing status, this imbalance may still limit generalizability to the broader working population, as the sample does not reflect the distribution of hearing ability in the general workforce."

8. **Measurement limitations of key constructs**

Issue: Job demands are measured with a five-item psychological demands subscale; supervisor support uses a four-item subscale of the Dutch JCQ. The authors note that the JCQ focuses on emotional support and may miss instrumental support.
Impact: Measurement limitations could attenuate or misrepresent moderation effects (matching principle).
Recommendation: Acknowledge and, if possible, conduct secondary analyses using alternative operationalizations (e.g., separate subscales for emotional vs instrumental support, if data exist). Consider discussing SAWSI or other measures for future work.

Response: We acknowledged in the Discussion that the JCQ supervisor support scale primarily reflects emotional support and does not capture instrumental support, which may have attenuated moderation effects in light of the matching principle. We also explicitly refer to the SAWSI as a measure that distinguishes emotional and instrumental forms of supervisor support and may provide a more nuanced assessment in future work. Unfortunately, the present dataset did not include a separate measure of instrumental supervisor support, which means that secondary analyses based on alternative operationalizations were not feasible. We have now made this explicit in the Discussion to avoid misunderstanding.

Adjustment in manuscript, page 14, line number 386: "In the present dataset, no separate measure of instrumental supervisor support was available, which precluded additional secondary analyses using alternative operationalizations."

9. **Matching principle and specificity of demands/support**

Issue: The authors discuss the matching principle but the study did not distinguish types of job demands (e.g., quantitative vs cognitive) or types of supervisor support.
Impact: The moderation may be stronger for certain demands or support types; aggregated measures could mask heterogeneity.
Recommendation: If data permit, perform exploratory analyses by subgrouping job demands (e.g., time pressure vs workload) and examine whether the buffering effect varies by demand type. Emphasize this as a direction for future work.

Response: We agree that the matching principle implies that moderation effects may differ across more specific types of job demands and supervisor support. As noted in the Discussion, the JCQ supervisor support scale primarily reflects emotional support, and our psychological job demands measure captures an aggregate construct rather than differentiating between specific demand subtypes. The present dataset did not include separate indicators of instrumental supervisor support or distinct categories of job demands, which precluded additional exploratory analyses of differential moderation effects.
We have now clarified this more explicitly in the Discussion and emphasized that future studies using more fine-grained assessments of both demands and supervisor support would be better able to test the matching principle in detail.

Adjustment in manuscript, page 14, line number 388: "Similarly, psychological job demands were assessed as an aggregate construct and did not allow separate examination of distinct demand types (e.g. quantitative vs cognitive workload). This means that

potential heterogeneity in the buffering effect across different kinds of job demands could not be examined.”

10. **Handling of missing data and attrition**

Issue: Excluded participants differed on several characteristics; the analysis uses a complete-case approach (n = 989 with 1,858 observations) from an eligible pool of larger size.
Impact: Potential selection bias and reduced generalizability.
Recommendation: Describe missing data patterns and consider multiple imputation or mixed-model advantages that handle missingness under MAR. Include a sensitivity analysis comparing complete-case vs imputed results.

Response: We examined missing data patterns in detail. Missingness mainly concerned the JCQ subscales and the 4DSQ distress score rather than covariates. Overall, 87 out of 1,076 eligible working participants (8.2%) had incomplete data on one or more model variables and were therefore excluded. Descriptively, excluded participants tended to be older and more often highly educated and permanently employed than those included in the analytic sample, while distributions of sex, hearing impairment and chronic disease appeared similar. Given the relatively low proportion of excluded participants (<10%), and the fact that missingness primarily affected exposure and outcome variables, a complete-case approach was considered acceptable. This is consistent with methodological guidance indicating that multiple imputation is not always required when the proportion of incomplete cases is small(4).

Adjustments in manuscript:

- Page 4, line number 135: “Participants who owned a business (n = 31) and those with incomplete data (n = 87, 8.2%) were excluded. Missing data predominantly concerned the JCQ and 4DSQ scales across waves; missingness in covariates was rare (<3%).”

- Page 5, line number 141: “As missingness mainly concerned the psychosocial work variables and distress scores, and the proportion of excluded participants was below 10%, a complete-case approach was considered acceptable (20).”

11. **STROBE/REDCAP-type reporting completeness**

Issue: The manuscript includes many elements well, but reporting could be strengthened in key areas (flow diagram, missing data details beyond the baseline).
Impact: Reviewers may note gaps in reporting standards.
Recommendation: Ensure a clear flow diagram with numbers at each stage (eligibility, inclusion, retention) across waves, and provide a table of missing data by variable and wave. Align with STROBE/MG guidelines explicitly in the methods/results sections.

Response: We appreciate the reviewer’s emphasis on transparent reporting. We followed the STROBE guidelines throughout the manuscript and Figure 1 provides a flow diagram detailing eligibility, exclusions and the final analytic sample. Furthermore, descriptive

comparisons between included and excluded participants based on missingness were provided in the text of the method section.
Because our analyses estimate overall mixed-effects associations rather than longitudinal change or attrition, we did not consider it necessary to present retention per wave. Nevertheless, we have now clarified the nature and extent of missing data more explicitly in the Participants section (see previous comment).

Reviewer 2 – Minor Comments

1. **Abstract clarity**

Include the study design term (e.g., "longitudinal cohort study with mixed-effects modeling") in the abstract's design line for quick indexing.

Response: We have revised the "Design" sentence in the abstract to clearly specify the design and analytic approach.

2. **Consistency of terminology**

Use consistent terminology for distress (4DSQ Distress) and ensure that "distress" vs "stress-related symptoms" aren't conflated in the narrative.

Response: We agree that conceptual clarity is important. In the Introduction, we already define "distress" at the outset as a non-specific psychological state operationalized by symptoms such as worry, tension, fatigue and concentration problems. Throughout the manuscript, we use the term "distress" consistently to refer to this construct. Alternative terms such as "stress-related illness" are only used when referring to prior studies from the literature that employed those labels. We believe this preserves conceptual consistency while remaining faithful to the terminology used in the cited literature. In line with this, the discussion explicitly notes that heterogeneous terminology across studies complicates comparison of findings and interpretation of evidence (page 11-12, line number 309).

3. **Figures and tables readability**

Some figures reference Model 2 and observed ranges; ensure captions clearly explain what is plotted (axes, units, SD units).
Add axis labels with real units and annotate effect sizes in the figure or include a supplementary figure with raw data ranges.

Response: To improve clarity, we have revised the figure caption to explicitly describe the axes, original scale units, SD-referencing and model specification. Raw distress scores are presented on their original scale in figure 2 and the raw ranges of the independent variables are now explicitly described in the caption.

Adjustment in manuscript, legend of Figure 2:
"Predicted 4DSQ distress scores (range 0–32) from Model 2 are shown across the observed range of centered JCQ job demands (raw scale range 12-48) and were centered at the grand mean before analysis. Lines represent estimated distress at low (–1 SD) and high (+1 SD) levels of JCQ supervisor support (range 4–16). Predicted values are adjusted for: age

(set at the sample mean), sex (male), educational level (intermediate), hearing status (no impairment), chronic disease (no), and contract type (permanent). Shaded areas indicate 95% confidence intervals. Predicted values are plotted only within the observed range of centered job demands (–18.9 to +17.1).”

4. **Reporting of interaction**

Report the simple slopes at representative values of supervisor support (e.g., low, mean, high) with Cis to aid interpretation, in addition to the stratified analysis.

Response: As recommended, the manuscript reports the estimated simple slopes of job demands at different levels of supervisor support. In Model 2, the regression coefficient for job demands represents the simple slope at the *mean* level of supervisor support, because supervisor support was centered at the grand mean prior to analysis. In addition, we present the simple slopes at low (–1 SD) and high (+1 SD) levels of supervisor support, each with 95% confidence intervals and p-values, and these are visualized in Figure 2 using marginal effects plots. To ensure this is also transparent to readers, we have now made this explicit in the results section.

Adjustment in manuscript, page 9, line 273:
“Because supervisor support was centered at the grand mean, the main effect of job demands in Model 2 represents the simple slope at the mean level of supervisor support. In addition, we present the simple slopes at low (–1 SD) and high (+1 SD) supervisor support, each with 95% confidence intervals and p-values.”

5. **Statistical software details**

They used R, lme4, and ggeffects. Consider providing code snippets or linking to an accessible repository (if allowed by data sharing policies) to enhance reproducibility.

Response: Because NL-SH data are only available under controlled access agreements, the dataset itself cannot be made publicly available.

To support transparency and reproducibility within the constraints of this data-sharing policy, we have now provided a public OSF repository containing the complete R code used for:
(1) estimating intraclass correlation coefficients (ICCs),
(2) specifying and fitting the mixed-effects models, and
(3) computing and visualizing marginal effects (including the simple slopes interaction plot).

This repository is openly accessible at: https://osf.io/rtnda
It contains only code; no data are included.

Adjustment made in manuscript, page 18, line 561: The link to the repository is added in the data availability statement in the manuscript.

6. **Data availability and ethics**

Data availability statement is present; ensure that documentation for data access (application process) is precise. Confirm ethics approvals across waves if applicable.

Response: The ethical approval section in text was updated.

Change in manuscript, page 4, line number 126:
"Baseline and 5-year wave ethical approval for the NL-SH were granted by the Medical Ethical Committee of the VU University Medical Center (now: Amsterdam UMC; 2006/83; NL12015.029.06). As of 2016 this committee decided amendments (including follow-up waves) are exempt from ethics approval."

7. **Table 1 (descriptive characteristics)**

Include p-values for group comparisons (if any) and/or standardized differences to assess balance, particularly for key covariates.

Response: Table 1 is intended to provide a descriptive overview of the analysis sample at the first eligible measurement occasion. Because this study does not compare predefined exposure groups or treatment arms, no hypothesis testing of baseline characteristics was performed. In line with the STROBE Explanation and Elaboration, descriptive tables should summarize participant characteristics and potential confounders, and "significance tests should be avoided in descriptive tables" (5). Adding p-values or standardized differences would therefore imply inferential group comparisons that are not part of the study design and are not recommended for observational cohort descriptions. Table 1 was therefore retained as a descriptive summary.

8. **Multicollinearity discussion**

Provide VIFs for the full model (including the interaction term). Explain any tolerance concerns or how collinearity was handled when interpreting the interaction.

Response: We recalculated VIFs for the full model including the interaction term; all VIFs remained < 1.3 (range 1.01–1.22), confirming absence of multicollinearity.

Adjustment in manuscript, page 7, line number 231:
"Multicollinearity was assessed using variance inflation factors (VIFs) based both an equivalent linear model and the full model including interaction terms. All VIFs were below 1.3, indicating no concerns regarding multicollinearity between the psychosocial work variables."

9. **Limitations expansion**

Add a brief note on potential reverse causation (e.g., distress influencing perceived supervisor support or perceived job demands) and how the study design mitigates or cannot mitigate this.

Response: To address the concern of the reviewer about reverse causation more explicitly, we now added that, given the self-reported and cross-sectional nature of the measurements within waves, higher distress may itself influence perceived job demands

and perceived supervisor support, and that this potential reverse causation cannot be ruled out

Adjustment in manuscript, page 12, line number 346:
"Moreover, because all variables were assessed by self-report at the same time point, higher distress may also influence how employees perceive their work environment, for example by increasing perceived job demands or lowering perceived supervisor support. This potential reverse causation cannot be ruled out given the design"

10. **Practical implications**

Provide concrete recommendations for workplaces (e.g., supervisor training components, documentation of supportive practices) and outline how organizations might monitor these variables in practice.

Response: Practical implications for workplace policy and practice are addressed in the *Implications* section (page 14). We describe how supportive supervisory behavior can be strengthened within organizations, provide examples of relevant workplace interventions, and discuss how participatory approaches may facilitate tailored problem-solving around job demands.

References

1. Twisk JW. Applied longitudinal data analysis for epidemiology: a practical guide: cambridge university press; 2013.
2. Terluin B. Four-dimensional symptom questionnaire (4DSQ). Encyclopedia of Quality of Life and Well-Being Research: Springer; 2021. p. 1-3.
3. Terluin B. De vierdimensionale klachtenlijst (4DKL). Een vragenlijst voor het meten van distress, depressie, angst en somatisatie [The four-dimensional symptom questionnaire (4DSQ) A questionnaire to measure distress, depression, anxiety, and somatization] Huisarts & Wetenschap. 1996;39(12):538-47.
4. Austin PC, White IR, Lee DS, van Buuren S. Missing data in clinical research: a tutorial on multiple imputation. Canadian Journal of Cardiology. 2021;37(9):1322-31.
5. Vandenbroucke JP, von Elm E, Altman DG, Gøtzsche PC, Mulrow CD, Pocock SJ, et al. Strengthening the Reporting of Observational Studies in Epidemiology (STROBE): Explanation and elaboration. International Journal of Surgery. 2014;12(12):1500-24.

VERSION 2 - REVIEW

**Reviewer**	**1**
**Name**	**Vitturi, Bruno**
**Affiliation**	**University of Genoa, DISSAL**

Date **21-Jan-2026**

COI

No further comment

Reviewer **2**

Name **Poursadeqiyan, Mohsen**

Affiliation **Ardabil University of Medical Sciences**

Date **28-Jan-2026**

COI

---

dont accept.

Reviewer **3**

Name **Moinuddin, Mohammed**

Affiliation **University of Lancashire, School of Medicine and Dentistry**

Date **23-Feb-2026**

COI

This is an interesting piece of work, which is well written, and the statistical analysis is thought in the right direction, and the output is presented well. For further transparency and clinical meaningfulness, some clarifications are required. My observations are below.

The manuscript reported that the distress score was moderately right-skewed. Did the authors test any alternative models, such as Gemma or Poisson regression?

The conclusions are heavily based on statistical significance (p-value based) and the magnitude of the beta coefficients is small. The authors claimed that they were modest. I wonder if the authors should have checked if any study reported a clinically meaningful difference for the main indicators and compared the clinical relevance of the results.

In figure 2, the authors presented the interaction effect using fitted line. I would love to see the boxplots or point range plot with fitted mean with some error bars which will provide more insight from the implication point of view. In addition, a scatter plot of outcome versus exposure with loess-type smoothed curves, potentially stratified by the moderator, would be beneficial. The scatter plot will also support of the association were linear which justifies the use of linear model.

VERSION 2 - AUTHOR RESPONSE

Reviewer:
The manuscript reported that the distress score was moderately right-skewed. Did the authors test any alternative models, such as Gemma or Poisson regression?

Response:
Although the distress score showed moderate right-skewness, linear mixed models do not require the dependent variable itself to be normally distributed; rather, the assumption concerns the distribution of the residuals. We therefore inspected model diagnostics, including residual-versus-fitted plots and Q–Q plots. These indicated no major deviations from homoscedasticity and showed that the residuals were approximately normally distributed as mentioned in the manuscript under statistical analyses, page 7, lines 236-238. The diagnostic plots are included in this response for transparency.
We also considered the suggested alternative models. Poisson regression is typically applied to count outcomes, whereas the distress score represents a bounded psychometric scale rather than count data. Gamma models are generally used for strictly positive continuous outcomes without an upper bound, which does not correspond to the characteristics of the distress scale (range 0–32). Given these considerations, linear mixed modelling was considered the most appropriate analytical approach.

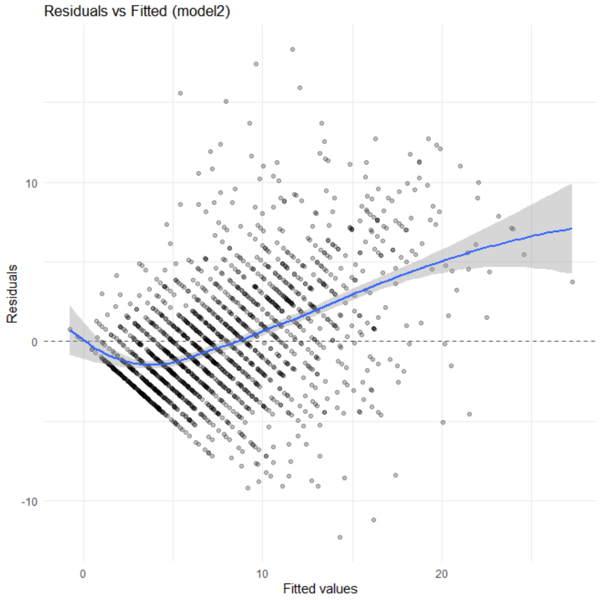

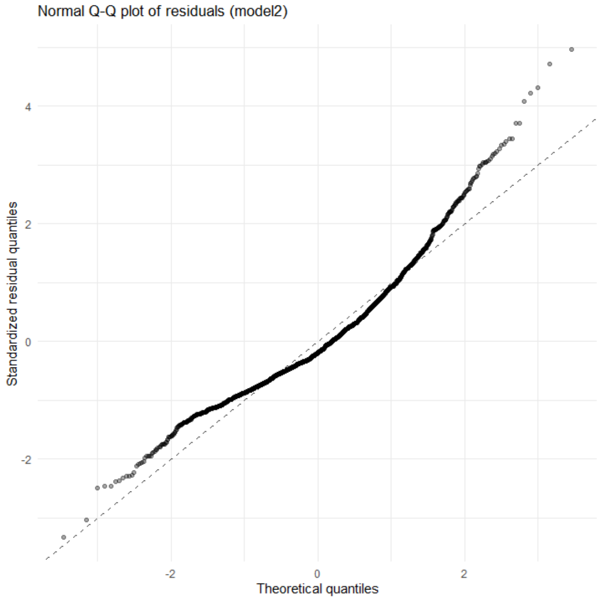

Reviewer:
The conclusions are heavily based on statistical significance (p-value based) and the magnitude of the beta coefficients is small. The authors claimed that they were modest. I wonder if the authors should have checked if any study reported a clinically meaningful difference for the main indicators and compared the clinical relevance of the results.

Response:
We agree that statistical significance should not be interpreted in isolation and that the practical relevance of observed associations deserves careful consideration. Previous research has shown that higher scores on the distress scale are meaningfully related to clinical outcomes; for example, each one-point increase in distress was associated with a 13% higher odds of receiving a psychosocial diagnosis in a primary care population (1). Also, a minimal important change (MIC) of 8.5 points has been reported for the 4DSQ distress scale (2). Although this provides additional context for interpreting changes in distress scores, the outcomes in that study differ methodologically from those examined in the present manuscript. The MIC value refers to within-person change over time, derived from differences in change scores between improved and unchanged patients. Although informative for interpreting individual improvement in longitudinal or intervention studies, such thresholds are not directly applicable to cross-sectional differences or regression-based associations between individuals, as examined in the present study.

To facilitate interpretation of the practical relevance of the results, we have therefore added predicted distress scores based on the interaction model. Predicted values ranged from 6.4 among employees with low job demands and high supervisor support to 10.1 among those with high job demands and low supervisor support. For transparency, the model-based predicted values and their 95% confidence intervals are shown below. These correspond to the values visualized in the supplementary point-range plot. Notably, predicted distress scores approached the threshold for moderately elevated distress (score >10) only among employees reporting both high job demands and low supervisor support. These values have been added to the Results section to provide a clearer indication of the magnitude of the observed interaction effect.

Job demands	***Supervisor support***	***Predicted distress***	***95% CI***
Low (-1 SD)	High (+1 SD)	6.4	5.8–6.9
Low (-1 SD)	Low (-1 SD)	7.1	6.5–7.8
High (+1 SD)	High (+1 SD)	8.3	7.7–8.9
High (+1 SD)	Low (-1 SD)	10.1	9.5-10.6

Changes in manuscript:

- Added to results, page 9, lines 279-283:
  "To facilitate interpretation of the magnitude of the interaction effect, predicted distress scores were calculated for combinations of low (–1 SD) and high (+1 SD) job demands and supervisor support. The predicted distress score ranged from 6.4 among employees with low job demands and high supervisor support to 10.1 among those with high job demands and low supervisor support."
- Nuanced main findings, page 11, lines 298-303:
  "In this study, higher job demands were associated with greater distress, whereas employees who perceived more supervisor support tended to report lower distress. In addition, supervisor support appeared to mitigate the positive association between job demands and distress. Although the magnitude of this moderating effect was modest, the findings suggest that supervisor support may function as a protective resource in demanding work contexts."

Reviewer:
In figure 2, the authors presented the interaction effect using fitted line. I would love to see the boxplots or point range plot with fitted mean with some error bars which will provide more insight from the implication point of view. In addition, a scatter plot of outcome versus exposure with loess-type smoothed curves,

potentially stratified by the moderator, would be beneficial. The scatter plot will also support of the association were linear which justifies the use of linear model.

Response:
Thank you for this constructive suggestion. To provide a more detailed visual representation of the data, we have added additional figures to this response:

Specifically, we included:

- boxplots illustrating the distribution of distress across levels of the main predictors,
- scatter plots of distress versus job demands with LOESS smoothing curves, stratified by supervisor support, and
- point-range plots showing estimated means with 95% confidence intervals.

These visualizations provide additional insight into the data distribution and support the linearity assumption underlying the applied regression models.

Boxplots
The boxplots illustrate the distribution of distress scores across tertiles of job demands and supervisor support. Distress tended to be higher in the higher job demands tertiles and lower among employees reporting higher levels of supervisor support. The distributions also show that distress scores were spread across the full scale range without extreme clustering, supporting the suitability of linear modelling.

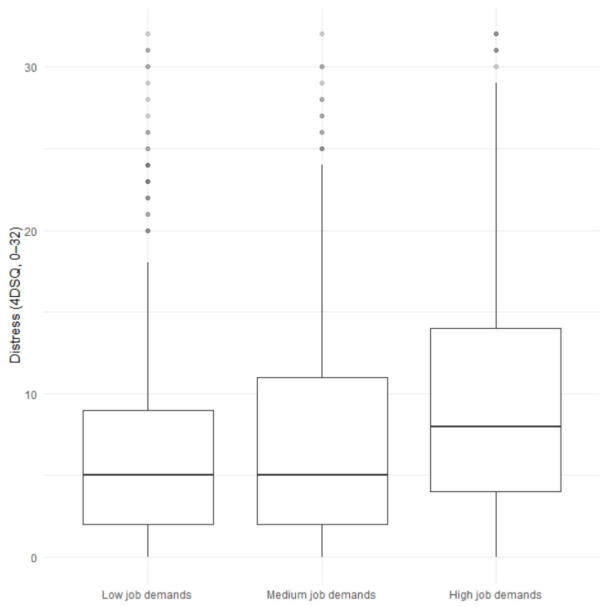

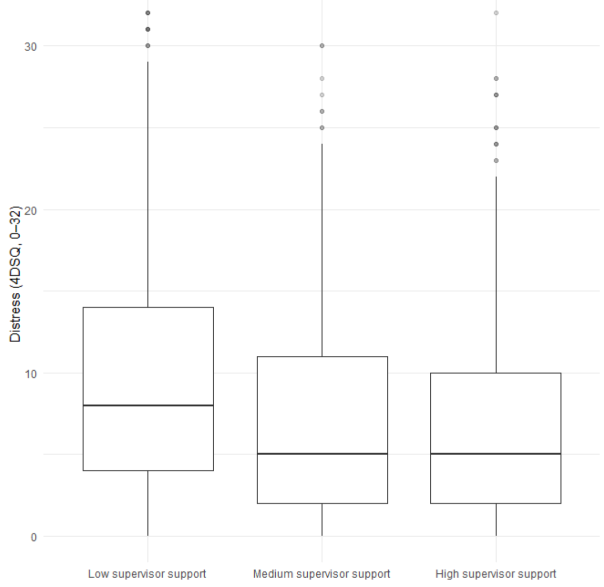

Scatterplots

The scatterplots display the association between job demands and distress, stratified by low (–1 SD) and high (+1 SD) supervisor support, with LOESS smoothing curves. In both strata, distress tended to increase with higher job demands, while the increase appeared steeper among employees reporting lower supervisor support. The LOESS curves suggested a generally positive association between job demands and distress, although some local curvature was visible, particularly in the high supervisor support group.

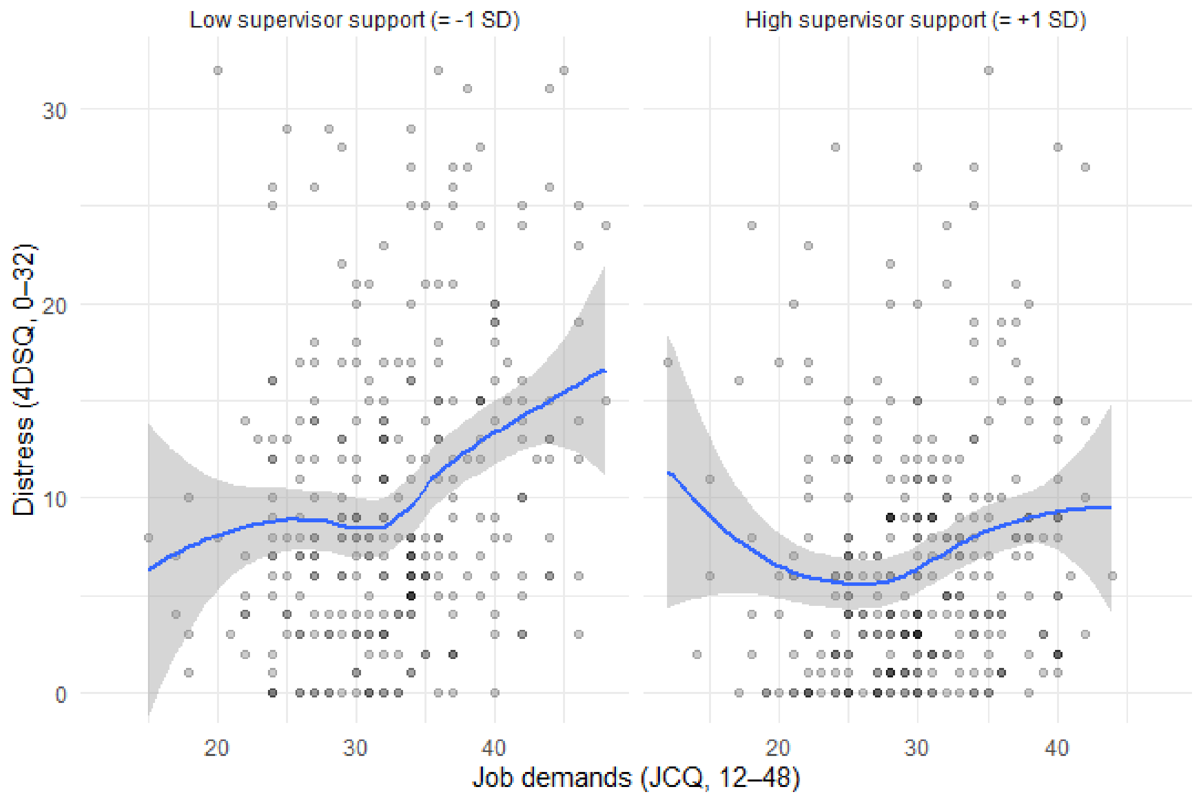

Point-range plots with a 95% CI

The point-range plot presents predicted distress scores with 95% confidence intervals for combinations of low and high job demands and supervisor support (±1 SD), based on the interaction model. Predicted distress ranged from approximately 6.4 among employees with low job demands and high supervisor support to approximately 10.1 among those with high job demands and low supervisor support. The figure illustrates that higher supervisor support attenuated the association between job demands and distress.

Two versions of the figure are provided: one showing the full scale range of the distress measure and a zoomed-in version focusing on the observed range of predicted values, to allow clearer visualization of the confidence intervals and differences between conditions.

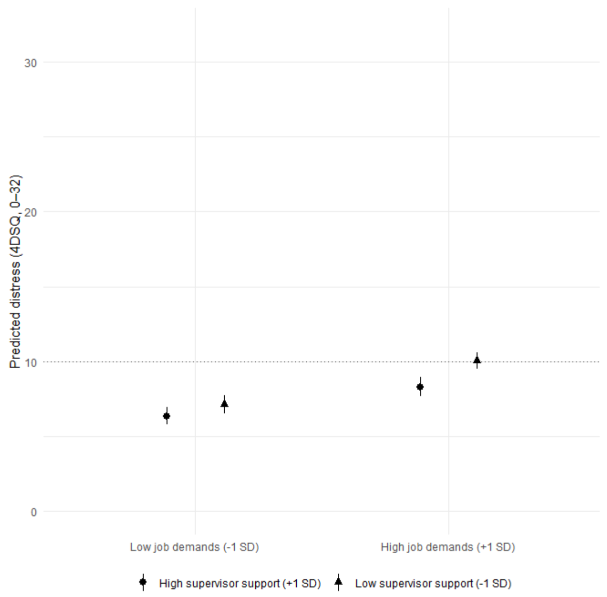

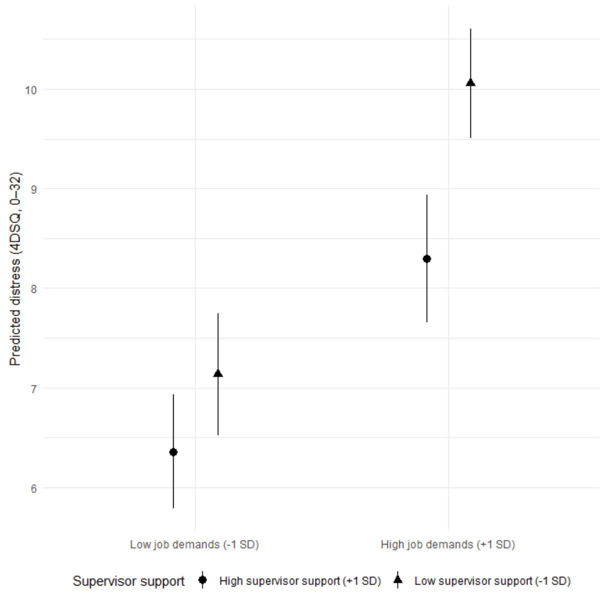

References:
1. Terluin B, van Marwijk HW, Adèr HJ, de Vet HC, Penninx BW, Hermens ML, et al. The Four-Dimensional Symptom Questionnaire (4DSQ): a validation study of a multidimensional self-report questionnaire to assess distress, depression, anxiety and somatization. BMC psychiatry. 2006;6(1):1-20.
2. Terluin B. Four-Dimensional Symptom Questionnaire (4DSQ). In: Maggino F, editor. Encyclopedia of Quality of Life and Well-Being Research. Cham: Springer International Publishing; 2023. p. 2585-8.
